# The role of blood–brain barrier dysfunction in cognitive impairments in bipolar disorder—a narrative review

**DOI:** 10.3389/fnhum.2025.1504575

**Published:** 2025-02-19

**Authors:** Caitlin E. Millett, Faria Monir, Pina Sanelli

**Affiliations:** ^1^Northwell, New Hyde Park, NY, United States; ^2^Feinstein Institutes of Medical Research, Manhasset, NY, United States; ^3^The Division of Psychiatric Research, Zucker Hillside Hospital, Glen Oaks, NY, United States; ^4^Department of Psychiatry, Donald and Barbara Zucker School of Medicine at Hofstra/Northwell, Hempstead, NY, United States

**Keywords:** cognitive deficits, biomarkers, neurovascular permeability, mood disorders, mania

## Abstract

Bipolar disorder (BD) is a chronic and debilitating mental illness affecting approximately 40 million people worldwide. Cognitive impairment is a core feature of BD, impacting daily functioning and persisting even during mood stability. Cognitive deficits are among the most reliable indicators of long-term functional outcomes in BD. Despite their significance, there are currently no widely available treatments targeting cognitive impairment in BD, largely due to our limited understanding of the underlying pathophysiology. A healthy blood–brain barrier (BBB) is essential for brain homeostasis, serving as a protective filter that restricts peripheral toxins, pathogens, and ions from entering the brain and disrupting neuronal function. Increased BBB permeability can allow harmful substances to infiltrate the brain, potentially leading to neuroinflammation, disrupted signaling, and damage to brain tissue, all of which may contribute to cognitive impairments in BD. Thus, BBB dysfunction could represent an upstream driver of cognitive impairment in BD, offering a potential target for disease-modifying interventions. This narrative review examined the evidence for the link between BBB permeability and cognitive deficits in BD. Our search yielded limited studies with mixed findings, highlighting the significant need for further research to explore this critical area and its potential for developing disease-modifying treatments.

## 1 Introduction

Bipolar disorder (BD) is a heterogeneous and disabling mental illness that affects approximately 40 million people around the globe (Lai et al., 2024). People with BD experience high rates of functional impairment, both occupationally and socially (Burdick et al., 2022; Léda-Rêgo et al., 2020). Cognitive deficits are among the largest contributors to functional impairment in individuals with BD (Depp et al., 2012), and exist across various domains with varying degrees of impairment (Bora and Özerdem, 2017; Burdick et al., 2011). An estimated 40–60% of patients with BD have cognitive impairments, many with severe, global impairments (Burdick et al., 2014). Severe cognitive impairment is associated with worse treatment response (Manove and Levy, 2010), episode relapse (Miskowiak et al., 2022), and lower quality of life (Cotrena et al., 2016). Despite the centrality of cognitive impairment in BD, there exists a substantial gap in our understanding of the etiology of cognitive deficits in BD. It is unknown whether cognitive changes follow a neurodevelopmental course, or also include a neuroprogressive component (Millett and Burdick, 2021).

Existing evidence regarding the effect of premorbid intelligence quotient (IQ) on subsequent BD diagnosis is mixed. A few large cohort studies suggest that premorbid IQ does not predict the subsequent onset of BD, unlike schizophrenia (Zammit et al., 2004). A large Swedish cohort study found that the risk of developing BD may vary based on specific premorbid cognitive domains: both high performance in arithmetic and low performance in visuospatial tasks have been identified as potential risk factors (Tiihonen et al., 2005).

Consistently, broad cognitive impairment is observed around the time of the first episode (Bora and Pantelis, 2015). The trajectory of cognitive performance after the onset of illness is poorly understood. Longitudinal studies in BD—most of which span fewer than 10 years (Van Rheenen et al., 2020; Samamé et al., 2022)—suggest that cognitive impairments in BD do not meaningfully worsen after illness onset (Van Rheenen et al., 2020). However, more (hypo)manic episodes have been linked with increased cortical thinning in BD in longitudinal neuroimaging studies (Abé et al., 2023; Abé et al., 2015). Furthermore, a large, longitudinal cohort study of patients with psychotic disorders supported the notion that the etiology of cognitive impairments has both neurodevelopmental and neuroprogressive underpinnings and that the neuroprogressive effects in BD patients with psychosis may occur at a slower rate relative to those with schizophrenia spectrum disorders (SSDs). The authors reported that in those with “other” psychotic disorders (*n* = 216)—including BD (*n* = 106), major depression (*n* = 43), substance-induced (*n* = 30), and not otherwise specified (*n* = 33)—there was a loss of one IQ point every 7 years versus one IQ point lost every 3 years for SSDs in the “declining phase” (Jonas et al., 2022) This is in line with some evidence indicating a decline in cognitive function in BD patients with psychosis over longer timeframes (20-year follow-up) (Fett et al., 2020). Evidence also suggests that people with BD are at higher risk of developing dementia than the general population and people with unipolar depression (Velosa et al., 2020).

These knowledge gaps have hindered efforts to develop interventions to slow or prevent the onset of cognitive dysfunction and decline. To date, no treatments are widely available to successfully ameliorate (or improve) cognitive impairment in BD (Solé et al., 2017; Burdick et al., 2012). Considering cognition’s impact on “everyday” function and mood episode onset and recurrence, successfully targeting cognition will have significant impacts on patients’ quality of life.

In recent years, the blood–brain barrier (BBB) has garnered increased attention in the psychiatric field. This is for good reason, as the BBB is the interface between the peripheral circulation and the central nervous system and is crucial to maintaining homeostasis by protecting the brain from bloodborne toxins, as well as tightly regulating the influx and efflux of oxygen, ions, nutrients, and water (Sweeney et al., 2019). Increased BBB permeability (BBBP) has been observed in various neuropsychiatric and neurodegenerative disorders (Sweeney et al., 2018; Najjar et al., 2013) and has been linked with cognitive decline progression (Puig-Pijoan et al., 2024) and worse functional outcomes after neural injury (Ivanidze et al., 2018). A burgeoning body of evidence suggests that there is increased BBBP in psychiatric disorders as well (Futtrup et al., 2020; Cheng et al., 2022). However, how BBBP is related, if at all, to the onset or progression of cognitive impairment in BD remains to be known. The aim of this narrative review is to summarize the evidence linking BBBP to cognitive performance/impairment in BD, current gaps in our knowledge, and where to go from here.

## 2 Methods

This narrative review, conducted in December 2024, investigated the relationship between BBB integrity and cognitive function in BD. Searches were performed in PubMed and APA PsychNet (PsycInfo). The first search broadly explored the association between BD and BBBP using the terms: (bipolar disorder OR psychosis OR psychotic OR mood disorder) AND (blood brain barrier OR BBB OR S100B OR DCE-MRI). The second search narrowed the focus to include cognitive measures, using the terms: (bipolar disorder OR psychosis OR psychotic OR mood disorder) AND (blood brain barrier OR BBB OR S100B OR DCE-MRI) AND (cognition OR cognitive OR neurocognitive OR executive function OR processing speed OR attention). After removing duplicates, 614 articles were screened for relevance based on title and abstract within Covidence. This initial screening yielded 89 articles to extract for full-text review. Inclusion criteria required direct measurement of both cognitive function (using a cognitive test) and at least one measurement of BBBP or function in individuals with BD. Ultimately, two articles met these criteria, summarized in Table 1.

**Table 1:** Articles directly exploring the association between markers of BBB function and cognition in bipolar disorder.

References	Methods (BBBP/cognition)	Participants	Main findings
Knorr et al. (2024)	S100B/SCIP-D, RAVLT, RBANS Digit Span, WAIS digit-letter substitution test, verbal fluency, TMT-A, TMT-B, CANTAB, DART	*N* = 85 BD	S100B was not significantly associated with any cognitive domain
Ottesen et al. (2020)	S100B/TMT-A, TMT-B, SCIP-D	*N* = 204 subjects (*N* = 31 BD)	Negative correlation between S100B and cognitive performance (global, executive function and working memory)

SCIP-D, Screen for Cognitive Impairment in Psychiatry—Danish Version (Purdon, 2005); RAVLT, Rey Auditory Verbal Learning Test (Schmidt, 1996; Rey, 1941); RBANS, Repeatable Battery for the Assessment of Neuropsychological Status (Randolph, 1998); WAIS, Wechsler Adult Intelligence Scale (Wechsler, 1997); TMT, Trail Making Test; CANTAB, Cambridge Neuropsychological Test Automated Battery (Cognition, 2018); DART, Danish Adult Reading Task (Crawford et al., 1987); UD, Unipolar depression.

## 3 Results

Given the essential role of the BBB in neurovascular coupling, nutrient uptake, toxin and waste removal, and ionic homeostasis in the CNS, a connection between BBBP and cognitive impairment may seem clear. Numerous studies have supported an association between BBBP and cognitive impairment in a variety of disorders and diseases, reviewed here (Sweeney et al., 2018). In contrast, there is a paucity of direct evidence linking BBBP to cognitive performance in BD. To our knowledge, there are only two studies that have directly examined this relationship so far (Table 1).

A cross-sectional study by Ottesen et al. (2020) identified 204 twins from Danish registries. They analyzed S100B in these participants across three groups: the “affected” group consisted of individuals with an affective disorder (27% were diagnosed BD, and the remainder had unipolar depression, UD); the second group was the high-risk co-twins of the affected group; the third group was unaffected low-risk twins. The authors did not find a significant difference in S100B across groups, nor did they find a difference in S100B between BD and UD participants (Ottesen et al., 2020). However, they found a significant association between S100B and cognitive performance in all participants (Ottesen et al., 2020). Specifically, they found that higher S100B (i.e., higher BBBP) was associated with poorer global cognition (SCIP Total), executive function, and working memory (Ottesen et al., 2020). A major limitation of this analysis was the diagnostic heterogeneity of the “affected” group—making definitive statements about the relationship between cognition and S100B in BD versus others impossible.

A more recent prospective, longitudinal study by Knorr et al. (2024) examined S100B solely in BD patients. This study aimed to assess the association between CSF and blood-based markers of neurodegeneration and cognitive performance in BD patients. The study recruited *N* = 85 patients with BD aged 18–69 while in remission, and they were followed for up to 1 year. In exploratory analyses, this study failed to find an association between S100B and cognitive performance across several domains (global cognition, verbal memory, executive function, psychomotor speed, and sustained attention) in patients (Knorr et al., 2024). However, they found a significant association between a biomarker of neurodegeneration (CSF Aβ42) and cognitive performance in patients (Knorr et al., 2024).

## 4 Discussion

Here, we present a narrative review of existing literature that examines cognitive performance and BBBP in individuals with BD. The literature search revealed two studies that met our criteria for inclusion—highlighting the paucity of published research on this topic in BD. The studies found disparate results, one indicating a possible relationship between BBBP and cognitive impairment and the other indicating no relationship between these variables. This discrepancy may be influenced by several factors. The study by Ottesen et al. (2020) used a transdiagnostic approach in their exploratory analyses—making diagnosis-specific conclusions difficult. The “affected” group was primarily composed of UD patients (72% of this group), so the effects may be primarily driven by them. This is supported by the study by Knorr et al. (2024), which analyzed data in only BD patients and found no significant associations between cognition and S100B. These negative and conflicting findings may have been influenced by a variety of factors, including mood state (patients were in remission at baseline) and the non-specific marker used to measure BBBP—S100B.

Existing research on BBBP in mood disorders thus far has utilized the “low hanging fruit” markers such as S100B—a calcium-binding protein localized to astrocytic end feet and a peripheral marker of BBB disruption—reviewed in a recent meta-analysis (Futtrup et al., 2020). Futtrup et al. (2020) showed that S100B is elevated across several diagnostic groups, including BD, SSDs, and MDD. The preponderance of evidence exists for SSDs, with 24 studies (*n* = 1,107 patients) included in this population. By comparison, very few studies (*n* = 4 studies with *n* = 142 patients) have examined this marker in BD. Intriguingly, most of the studies that looked at S100B in BD were in the manic phase (Tsai and Huang, 2017; Machado-Vieira et al., 2004; Andreazza et al., 2007). In fact, one very small meta-analysis has reported increased BBBP in manic BD specifically, supporting this theory (da Rosa et al., 2016). More research needs to be done to validate these initial findings. A very recent systematic review explored evidence for BBBP in BD specifically. Overall, there were 55 studies examining BBBP in BD, 38 of which reported higher BBBP and BD (Wakonigg Alonso et al., 2024). A total of 29 studies examined serum or CSF markers, and of those, 16 supported higher BBBP in BD across a range of markers, including S100B, matrix metalloproteinases (MMPs), and tight junction molecules, among others (Wakonigg Alonso et al., 2024). Only one study was identified, which looked specifically at Q_alb_ in BD and found it to be increased in BD (Zetterberg et al., 2014). Nearly all studies included in this systematic review used indirect markers, such as blood-based and CSF-based measures, and only one study used DCE-MRI (the reference standard) (Kamintsky et al., 2019). The diversity of measures used makes direct comparison difficult. Overall, these reviews support a link between increased BBBP and BD. However, there are limitations to using indirect measures of BBBP. Specifically, S100B is known to be highly expressed in astrocytic end feet. However, it has peripheral sources as well, including adipocytes (Michetti et al., 2019). This is a major confounder of many studies which do not statistically control for BMI, which tends to be elevated in BD patient populations. Similarly, the concentration of albumin in the CSF (which is normally low) could be higher not due to increased leakiness of brain barriers but because there is reduced volume of CSF (Asgari et al., 2017). It should be noted that increased albumin in the CSF is likely a marker of BCSFB leakiness, *not* BBB leakiness, although it is commonly reported as such in literature (Yakimov et al., 2024).

Only a handful of studies have directly measured BBBP using DCE-MRI in psychiatric patient populations. To our knowledge, only two studies have reported results using DCE-MRI in SSDs. One study by Cheng et al. (2022) observed increased K_trans_ in the thalamus. This is intriguing because the thalamus is implicated in the pathophysiology of psychosis. They also observed a positive correlation between K_trans_ and PANSS scores in patients. Another study by Moussiopoulou et al. (2023) observed elevated K_trans_ in SSD patients compared to controls in several brain regions, including the thalamus. However, they did not observe an association between K_trans_ and symptom severity on the PANSS or cognition. To our knowledge, there exists only one published study using DCE-MRI in patients with BD. Kamintsky et al. (2019) found increased BBBP in a subset of BD patients (10 of a total of 36 patients) which was associated with higher BMI, insulin resistance, and risk of cardiovascular disease. The authors also reported that higher K_trans_ was associated with a more severe course of illness in a retrospective analysis.

Literature on other neuropsychiatric disorders has supported a relationship between BBBP and cognitive/functional outcomes. A retrospective study by Ivanidze et al. (2018) found that in patients with sub-arachnoid hemorrhage (SAH, *n* = 22), a ROC curve analysis of four BBBP DCE-MRI parameters (K_trans_, Ve, PS, and K_ep_) had an area under the curve of 0.89 for prediction of modified Rankin scores 3–6 (i.e., BBBP parameters can predict more severe functional disability outcomes post-stroke). The authors concluded that BBBP parameters might have prognostic utility for stroke outcomes. A more recent prospective study by Puig-Pijoan et al. (2024) reported that among patients with dementias (*n* = 273), cognitive decline was predicted by higher BBBP (Q_alb_). They concluded that increased BBBP might contribute to clinical worsening in patients with dementia. Overall, these findings suggest that BBBP may have prognostic utility across neuropsychiatric disorders and highlight the need for similar approaches in BD. To date, no study in BD patients has examined the relationship between BBBP on cognitive outcomes using DCE-MRI.

This area is a burgeoning topic in the literature, and more research is necessary to advance this field. It should be noted that much more work has been done so far examining the interrelated features of neurophysiology, including glucose metabolism (e.g., FDG-PET) and cerebral blood flow (CBF), which can complement the existing data on BBBP. For example, the preponderance of published literature examining CBF has suggested that there is hypoperfusion in patients with BD during mood episodes (Toma et al., 2018). Considering the potential mechanistic links between BBBP and CBF—specifically, increased BBBP may result in reduced CBF or vice versa—it may be hypothesized that areas of the brain with reduced CBF may be excellent candidates for examining BBBP (Xu et al., 2022). Even so, based on the existing evidence, there is little data to support a significant relationship between BBBP and cognition in BD patients.

Parsing cognitive heterogeneity in BD may be important for elucidating the effect of BBBP on cognition. Clustering analyses have shown an approximately 40% of BD patients have global/widespread impairments across cognitive domains, while others are “selectively” impaired or “intact” (Burdick et al., 2014; Burdick and Millett, 2021). Given the heterogeneity and likely multifaceted causes of cognitive impairment, there is a demand for more personalized treatment strategies. Despite the considerable research on pharmacological interventions (Miskowiak et al., 2016; Miskowiak et al., 2018), few have emerged as effective, widely available treatment options for cognitive impairments in BD. Cognitive remediation/training is the most supported intervention for addressing these impairments (Miskowiak et al., 2017; Xu et al., 2022). A multifaceted approach utilizing both psychological and pharmacological interventions may ultimately be most beneficial for effective amelioration of cognitive impairments in BD. If BBBP is shown to be a driver of cognitive impairments in some patients, vascular stabilizing medications may be useful adjuncts to more traditional treatments for mood and cognitive symptoms in BD (Hashimoto et al., 2021).

**Figure 1:**
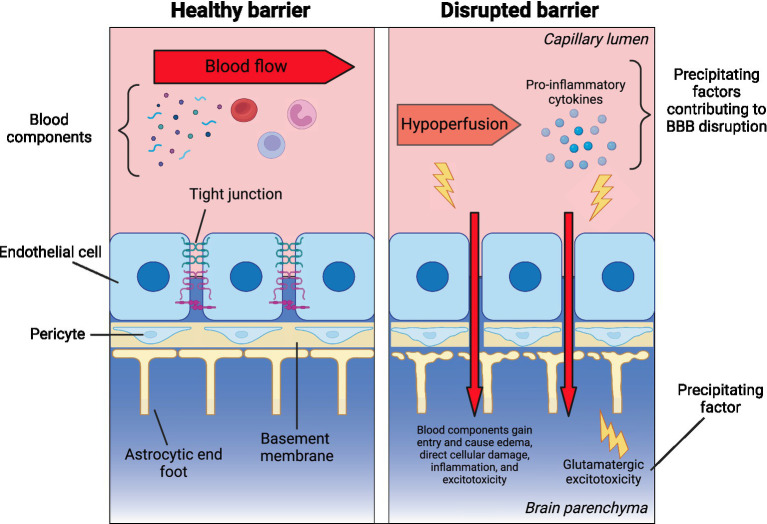
Theoretical framework: blood–brain barrier disruption in bipolar disorder. The left panel illustrates the key components of a healthy blood–brain barrier (BBB), separating the peripheral circulation and CNS. These include endothelial cells with tight junctions, the basement membrane, pericytes, and astrocytic end feet. The right panel presents a theoretical model of factors that may increase BBB permeability (BBBP), potentially leading to secondary damage from peripheral components entering the CNS through a leaky barrier. Created in BioRender. Millett, C. (2025), https://BioRender.com/j89y763.

Based on the limited existing evidence, it can be hypothesized that during mood episodes, several processes—such as increased pro-inflammatory cytokines, decreased CBF, and excitotoxicity in the CNS—may disrupt the BBB (Figure 1). These disruptions in the BBB may contribute to cognitive impairment through several mechanisms. When the BBB is compromised, the neurovascular unit (NVU) becomes permeable to infiltration by toxins and peripheral proteins, leading to brain tissue damage, edema, and disruptions in cerebral blood flow (CBF) and neurovascular coupling (Stanimirovic and Friedman, 2012). These changes can further exacerbate neuronal damage and cause inflammation in the CNS.

In BD, hypoperfusion—particularly in the frontal cortices—has been linked to cognitive deficits, as these areas are critical for executive functions and emotion regulation (Toma et al., 2018). Additionally, elevated levels of circulating pro-inflammatory markers, which are commonly observed in BD, especially during acute episodes (Fernandes et al., 2016; Goldsmith et al., 2016), may drive BBB dysfunction and amplify neuroinflammatory responses, worsening cognitive outcomes. While the role of glutamate remains complex, evidence from magnetic resonance spectroscopy (MRS) studies suggests glutamatergic dysregulation exists in the anterior cingulate cortex (ACC), particularly during depressive episodes in BD (Ino et al., 2023). This complex interplay of vascular, inflammatory, and excitotoxic factors within disrupted BBB regions may collectively impair cognitive function in BD. These processes could theoretically impair neuronal firing and cognitive function, potentially persisting long after the initial insult. However, these ideas are currently theoretical and require testing in BD patients during acute illness.

In conclusion, the role of BBBP in the onset and progression of cognitive deficits in BD remains largely unknown. A critical next step is to employ neuroimaging techniques like DCE-MRI longitudinally to assess functional BBB changes in patients quantitatively. This should be done in conjunction with longitudinal cognitive and clinical assessments to develop causal models of cognitive dysfunction in BD. One particularly important area of inquiry is the relationship between BBBP, and the cognitive impairments frequently observed around a patient’s first episode. More granular investigation of this timeframe, including before illness onset, could significantly advance our understanding of the disorder’s trajectory. Furthermore, broadening the scope of investigation to encompass dysfunction in the BCSFB will provide a more complete picture. A deeper understanding of both the qualitative and quantitative features of barrier dysfunction, combined with advancements in neuromodulation techniques and their effects on the brain’s barriers, holds significant therapeutic potential. For instance, if increased BBBP is confirmed as a key driver of any aspect of BD pathophysiology, interventions aimed at strengthening the barrier could be prioritized. Conversely, if barrier dysfunction hinders drug delivery to the central nervous system, neuromodulation strategies that transiently open the barrier might enhance treatment efficacy.

## Data Availability

The original contributions presented in the study are included in the article/supplementary material, further inquiries can be directed to the corresponding author.
